# Long‐Term Safety and Efficacy of Efgartigimod PH20 in Chronic Inflammatory Demyelinating Polyradiculoneuropathy: ADHERE/ADHERE+ Trial Interim Analysis

**DOI:** 10.1111/jns.70140

**Published:** 2026-07-16

**Authors:** Jeffrey A. Allen, Geoffrey Istas, Satoshi Kuwabara, Trevor Mole, Luis Querol, Simon Rinaldi, Mark Stettner, Benjamin Van Hoorick, Richard A. Lewis

**Affiliations:** ^1^ University of Minnesota Minneapolis Minnesota USA; ^2^ argenx Ghent Belgium; ^3^ Chiba University Chiba Japan; ^4^ Universitat Autònoma de Barcelona Barcelona Spain; ^5^ Centro De Investigación Biomédica en Red en Enfermedades Raras (CIBERER) Madrid Spain; ^6^ University of Oxford Oxford UK; ^7^ University Medicine Essen Essen Germany; ^8^ Cedars‐Sinai Medical Center California Los Angeles USA

**Keywords:** chronic inflammatory demyelinating polyradiculoneuropathy, efgartigimod, FcRn, INCAT, I‐RODS

## Abstract

**Background and Aims:**

In ADHERE, subcutaneous efgartigimod PH20 (1000 mg once weekly) was effective and well tolerated in participants with chronic inflammatory demyelinating polyradiculoneuropathy (CIDP). ADHERE+ is an open‐label extension of ADHERE assessing long‐term safety and efficacy.

**Methods:**

Eligible participants from ADHERE run‐in period (prior CIDP treatments discontinued), stage A (open‐label efgartigimod), and stage B (stage A responders randomized to placebo or efgartigimod) could roll over to ADHERE+ and receive efgartigimod. The primary outcome was to assess long‐term safety and tolerability. Efficacy outcomes evaluated adjusted Inflammatory Neuropathy Cause and Treatment (aINCAT) score, Inflammatory Rasch‐built Overall Disability Scale (I‐RODS) score, and mean grip strength (GS) from ADHERE run‐in baseline through ADHERE+ week 36.

**Results:**

At interim data cut‐off (February 16, 2024), 228/229 eligible participants advanced to ADHERE+. Prolonged efgartigimod exposure did not increase the incidence (*n* = 171/228 [75.0%]) or severity of TEAEs (grade ≥ 3 TEAEs, *n* = 41/228 [18.0%]); regardless of immunoglobulin G level, there was a low incidence of infections. Stage A responders reported long‐term clinically meaningful improvements in mean aINCAT (decreased by 1.2 points), I‐RODS centile metric (increase of 8.8 points), and GS scores (increase of 17.5 kPa) from run‐in baseline to ADHERE+ week 36, irrespective of stage B treatment.

**Interpretation:**

Interim results from ADHERE+ indicate long‐term efgartigimod PH20 treatment in participants with CIDP was well tolerated (maximum exposure = 187.3 weeks; mean (SD) treatment duration = 58.0 [33.8] weeks). Clinically meaningful improvements in disability and strength were observed across assessments, regardless of stage B treatment or prior treatment status, with greater improvements seen over time.

## 1 Introduction

Chronic inflammatory demyelinating polyradiculoneuropathy (CIDP) is an inflammatory peripheral neuropathy characterized by muscle weakness and sensory disturbance [1, 2, 3, 4, 5]. Traditional treatments for individuals with CIDP include corticosteroids, plasma exchange, or immunoglobulin (Ig) [5]. While these therapies are effective in the majority of patients, some patients fail to respond [5, 6]; additionally, the associated side effects and risks impose a considerable burden on some patients [5, 6, 7]. Combination treatment approaches have been explored in CIDP with the goal of maximizing therapeutic response; however, increased thromboembolic risk was associated with combined intravenous Ig (IVIg) and corticosteroid treatment in the OPTIC trial, leading to premature termination of the trial and highlighting the need to consider alternative approaches for patients with inadequate response to traditional therapies [8]. There remains an unmet need for efficacious, well‐tolerated, sustained, and convenient treatment options appropriate for long‐term use.

Efgartigimod is a human immunoglobulin G (IgG) antibody Fc fragment engineered for increased affinity for the neonatal Fc receptor (FcRn) compared with endogenous IgG, and is uniquely composed of the only part of the IgG antibody that normally binds FcRn [9, 10, 11]. Efgartigimod selectively reduces IgG by blocking FcRn‐mediated IgG recycling without impacting antibody production, reducing albumin levels, or increasing low‐density lipoprotein cholesterol [9, 10, 11, 12]. Subcutaneous (SC) efgartigimod PH20 is a coformulation of efgartigimod and recombinant human hyaluronidase PH20 [13]. Efgartigimod is approved for treating adults with CIDP in the United States, European Union, Japan, Canada, Israel, and China [14, 15]; it is also approved in multiple countries and regions for treating generalized myasthenia gravis in individuals who are anti‐acetylcholine receptor antibody–positive, and in Japan for primary immune thrombocytopenia [14]. Recently, prefilled syringes of efgartigimod PH20 SC became available, providing patients with the opportunity for self‐administration over 20–30 s at home [16].

In the multistage, double‐blind, placebo‐controlled ADHERE trial (NCT04281472), efgartigimod PH20 SC (1000 mg once weekly) reduced the risk of relapse and led to clinically meaningful improvements in functional ability, daily activity, and grip strength (GS) versus placebo, and was well tolerated in participants with CIDP [17]. ADHERE+ is currently investigating the long‐term safety, tolerability, and efficacy of efgartigimod PH20 in CIDP. Here, we report the results of an interim analysis (data cut‐off: February 16, 2024).

## 2 Materials and Methods

### 2.1 Study Design and Participants

ADHERE+ is an ongoing, global, open‐label, long‐term extension of the ADHERE trial (Figure S1), and eligibility criteria for ADHERE have previously been published [17]. Briefly, adults aged ≥ 18 years with CIDP (definite or probable) who had a CIDP Disease Activity Status score ≥ 2 at screening, and an Inflammatory Neuropathy Cause and Treatment (INCAT) score ≥ 2 (a score of 2 had to be exclusively from leg disability) were eligible for inclusion. Eligibility was confirmed when two adjudicators from the Diagnostic Adjudication Committee (DAC) independently confirmed a participant had definite or probable CIDP (as per the 2010 European Federation of Neurological Societies/Peripheral Nerve Society criteria [18]); in cases of disagreement, the DAC Chair (or designee) provided final determination. As such, ADHERE+ included participants from the ADHERE run‐in period (during which prior CIDP treatments were discontinued to identify participants with active disease), stage A (participants received open‐label efgartigimod PH20 treatment) and stage B (stage A responders randomized to receive placebo or efgartigimod PH20 treatment). Participants in ADHERE who were eligible to roll over into ADHERE+ included those who had a clinical deterioration in stage B or had completed week 48 of stage B. Additionally, participants who were ongoing in the run‐in period or receiving treatment in stage A or stage B could roll over into ADHERE+ if they underwent early termination because sufficient events for the primary endpoint had been reached (i.e., 88th relapse) (Figure S1). Participants who were excluded from ADHERE+ included nonresponders in stage A before the primary endpoint (88th relapse) was reached; participants who discontinued stage A before 12 weeks for other reasons; or participants reporting use of prohibited medications.

ADHERE+ was conducted in accordance with the protocol and consensus ethical principles derived from international guidelines (i.e., the Declaration of Helsinki, Council for International Organizations of Medical Sciences International Ethical Guidelines, applicable International Council for Harmonization of Technical Requirements for Pharmaceuticals for Human Use Good Clinical Practice Guidelines) and other applicable laws and regulations. Institutional review boards or independent ethics committees at each participating site approved the ADHERE+ protocol. The trial was registered with ClinicalTrials.gov (NCT04280718) and the EU Clinical Trials Register (EudraCT 2019–003107‐35).

### 2.2 Treatment

During ADHERE+, participants rolling over from ADHERE were initiated on open‐label efgartigimod coformulated with recombinant human hyaluronidase PH20 (efgartigimod PH20) SC 1000 mg once weekly. Participants completing ≥ 24 weeks of once‐weekly dosing and with stable disease for ≥ 12 weeks were eligible to receive efgartigimod PH20 once every 2 weeks; participants stable for ≥ 24 weeks while receiving efgartigimod PH20 every 2 weeks were eligible to receive the dose once every 3 weeks. Initially, efgartigimod PH20 SC was administered by either trained staff or nurses at the study site, but after training and between the scheduled study visits, efgartigimod was administered by the participant (self‐administration) or caregiver, a home nurse, or a concierge service at the study site. ADHERE+ was planned in 48‐week treatment periods, and participants could complete the study after 1 or more 48‐week treatment periods.

### 2.3 Endpoints

The primary objective was to assess the long‐term safety and tolerability of efgartigimod PH20; primary outcome measure was the incidence of treatment‐emergent adverse events (TEAEs) and serious adverse events (SAEs). Long‐term treatment effect was assessed by the change from ADHERE+ baseline over time in adjusted Inflammatory Neuropathy Cause and Treatment (aINCAT) score, the 24‐item Inflammatory Rasch‐built Overall Disability Scale (I‐RODS) score, and mean GS assessed by Martin Vigorimeter. Given the differences in ADHERE stage B treatment protocol (i.e., efgartigimod vs. placebo), a post hoc analysis of efficacy outcomes was conducted from ADHERE run‐in baseline to ADHERE+ week 36 for a more comprehensive efficacy assessment. Post hoc efficacy data included change in aINCAT, I‐RODS and mean GS, longitudinal scores for aINCAT, I‐RODs, and GS by prior treatment group (IVIg/SCIg, corticosteroids, and off‐treatment) and disease restabilization (aINCAT scores improving to stage B baseline levels or better).

### 2.4 Assessments

In ADHERE+, adverse events (AEs) were monitored at study visits, and participants could contact the site to report AEs at any time; AEs were coded by System Organ Class and Preferred Term according to Medical Dictionary for Regulatory Activities Version 25.1 (September 2022). Longitudinal aINCAT, I‐RODS score, and mean GS were evaluated weekly in stage A and every 4 weeks in stage B in ADHERE, and at week 4, 12, then every 12 weeks in ADHERE+. Value thresholds representing minimal clinically important differences were aINCAT change of ≥ 1 point, I‐RODS change of ≥ 4 points, and mean GS of ≥ 8 kPa in either hand [5, 19].

### 2.5 Statistical Analysis

This interim analysis analyzed participant disposition, demographics, and disease characteristics, and safety analyses using the safety analysis set. Efficacy analyses were performed on the ADHERE modified intention‐to‐treat (mITT) analysis set, for whom run‐in baseline data were available. Event rates were defined as the number of events/participant‐year of follow‐up (PYFU; defined as the sum of the follow‐up times of all participants in the applicable period). Primary and secondary endpoints were summarized by treatment during ADHERE using descriptive statistics. No imputation of missing values was performed, and all assessments were based on observed cases.

Post hoc analyses included the occurrence of infections per PYFU by lowest recorded IgG level split by quartile and the occurrence of injection‐site reactions (ISRs) over time and by severity. Clinically meaningful improvements in aINCAT score, I‐RODS centile metric score, and dominant‐hand GS were assessed among stage A responders with run‐in baseline (prior CIDP treatments discontinued) values and ongoing on ADHERE+ at data cut‐off. Longitudinal aINCAT scores, I‐RODS, and GS were evaluated by prior treatment group (IVIg/SCIg, corticosteroids, and off‐treatment) in the mITT population. Disease restabilization (aINCAT scores improving to stage B baseline levels or better) was evaluated in participants who experienced disease relapse during stage B (a ≥ 1‐point increase in aINCAT compared with stage B baseline).

## 3 Results

This interim analysis includes data from 125 study sites that enrolled participants in Austria, Belgium, Bulgaria, China, Denmark, France, Georgia, Germany, Israel, Italy, Japan, the Netherlands, Poland, Romania, Russian Federation, Serbia, Spain, Taiwan, Turkey, Ukraine, the United Kingdom, and the United States.

### 3.1 Participants

#### 3.1.1 Treatment Status

Of the 322 participants who entered ADHERE stage A, 222 enrolled into ADHERE+. Additionally, seven participants enrolled in the open‐label extension study directly from the run‐in period. ADHERE participants who did not advance to ADHERE+ included those who were stage A nonresponders, participants who discontinued stage A before 12 weeks, or those who reported use of prohibited medications. Among the 229 participants enrolled in ADHERE+, 228 (99.6%) received ≥ 1 dose or part of a dose of efgartigimod PH20 SC; one participant did not receive efgartigimod PH20 and was excluded from further analyses (status at rollover, run‐in period/stage A: 29; efgartigimod PH20 group in stage B: 99; placebo group in stage B: 100). At data cut‐off in ADHERE+, mean (SD) treatment duration in ADHERE+ was 58.0 (33.8) weeks. Participants received on average 56.0 (SD, 32.3) injections of efgartigimod PH20, and adherence was high (mean [SD] treatment compliance 98.5% [5.0]) during ADHERE+. Among participants who received efgartigimod PH20 continuously (i.e., received efgartigimod PH20 in stage B and enrolled in ADHERE+), the maximum duration of exposure to efgartigimod PH20 SC throughout the study (i.e., ADHERE and ADHERE+) was 187.3 weeks and the mean exposure duration was 85.9 weeks.

At this interim analysis, efgartigimod PH20 treatment was ongoing in 174/228 (76.3%), completed (i.e., participants decided to not start a new study cycle) by 3/228 (1.3%), and discontinued by 51/228 (22.4%) participants (Figure S2). Seventeen participants (7.5%) discontinued treatment due to AEs; in 12 of these, AEs were unrelated to CIDP (worsening), and 9/12 were considered not/unlikely related to efgartigimod PH20 treatment or study design. Among participants who reported CIDP (worsening), 4/5 cases were deemed not/unlikely related to treatment. Of the seven participants who discontinued the study owing to lack of efficacy, all had relapsed (i.e., a ≥ 1‐point increase in aINCAT compared with stage B baseline) during ADHERE stage B before continuing into ADHERE+ (four participants received efgartigimod PH20 and three participants received placebo during stage B).

#### 3.1.2 Demographics and Characteristics

The baseline characteristics of ADHERE+ participants (assessed at screening in ADHERE) were broadly representative of the overall ADHERE stage A participants (Table 1). Although participants in ADHERE+ represented a subset of ADHERE (predominantly stage A responders), their baseline characteristics were similar to and comparable with the overall ADHERE stage A baseline population, particularly with respect to the initial severity of CIDP.

**TABLE 1:** Demographics and baseline characteristics in ADHERE stage A and the ADHERE+ open‐label extension study.

	ADHERE stage A (*N* = 322)	ADHERE+ (*N* = 228)
Scores shown were assessed at screening in ADHERE		
Age, year, mean (SD)	54.0 (13.9)	53.2 (14.1)
Sex at birth, male, *n* (%)	208 (64.6)	142 (62.3)
Time since diagnosis, years, mean (SD)	4.9 (6.1)	4.9 (5.6)
Typical CIDP diagnosis, *n* (%)	268 (83.2)	199 (87.3)
Unstable active disease (CDAS: 5),^a^ *n* (%)	197 (61.2)	151 (66.2)
Prior treatment (within past 6 months), *n* (%)		
Corticosteroids	63 (19.6)	51 (22.4)
Immunoglobulins (IVIg, SCIg)	165 (51.2)	104 (45.6)
Off‐treatment	94 (29.2)	73 (32.0)
Scores assessed at ADHERE stage A baseline for ADHERE+		
Total aINCAT score, mean (SD)^b^	4.6 (1.7)	4.5 (1.6)
I‐RODS score, mean (SD)^c^	40.1 (14.7)	41.2 (15.4)
Grip strength (dominant hand), kPa, mean (SD)^d^	38.5 (24.2)	39.0 (23.6)

Abbreviations: aINCAT: adjusted Inflammatory Neuropathy Cause and Treatment; CDAS: chronic inflammatory demyelinating polyneuropathy disease activity status; CIDP: chronic inflammatory demyelinating polyradiculoneuropathy; I‐RODS: Inflammatory Rasch‐built Overall Disability Scale; IVIg: intravenous immunoglobulin; kPa: kilopascal; PH20: recombinant human hyaluronidase PH20; SC: subcutaneous; SCIg: subcutaneous immunoglobulin; SD: standard deviation.

^a^ Unstable active disease was defined as abnormal examination with progressive or relapsing course.

^b^ aINCAT assesses arm and leg physical functioning, with scores ranging between 0 and 10; 0 = no disability; 10 = maximum disability.

^c^ I‐RODS scores are based on a 24‐item questionnaire assessing physical and social activities, scored as 0 (impossible to perform), 1 (performed with difficulty), and 2 (easily performed).

^d^ Grip strength scores in the non‐dominant hand were similar.

### 3.2 Long‐Term Safety and Tolerability

TEAEs were reported in 171/228 (75.0%; 3.1 events/PYFU) participants, with grade ≥ 3 TEAEs reported by 41 (18.0%; 0.31 events/PYFU) participants (Table 2). TEAEs leading to treatment discontinuation occurred in 18 (7.9%; 0.14 events/PYFU) participants; events leading to discontinuation in two or more participants were CIDP (worsening) (*n* = 5; 0.027 events/PYFU) and breast cancer (*n* = 2; 0.008 events/PYFU). No rescue therapy was specified in the study protocol. Regardless of the reason for withdrawal, participants who exited the study were managed according to the local standard of care at the discretion of the treating physician. Treatment‐related AEs according to the investigator occurred in 70/228 (30.7%; 0.74 events/PYFU) participants. None of the treatment‐related AEs by preferred term occurred in ≥ 5% of participants, except for upper respiratory tract infection (13/228 [5.7%] participants). The most common TEAEs were COVID‐19 (37/228 [16.2%]; 0.14 events/PYFU) and upper respiratory tract infection (24/228 [10.5%]; 0.15 events/PYFU) (Table 2).

**TABLE 2:** Safety and tolerability of efgartigimod PH20 SC in the ADHERE+ open‐label extension study.^a^

*n* (%) [event rate^b^]	Efgartigimod PH20 SC (*N* = 228; PYFU = 263.0)
Overview of adverse events	
Any TEAE	171 (75.0) [3.10]
Any grade ≥ 3 TEAE	41 (18.0) [0.31]
Discontinued due to TEAEs^c^	18 (7.9) [0.14]
Treatment‐related AEs	70 (30.7) [0.74]
Any SAE	35 (15.4) [0.25]
Treatment‐related SAEs	4 (1.8) [0.02]
Deaths^d^	2 (0.9) [< 0.01]
Any ISRs^e^	29 (12.7) [0.27]
Most common TEAEs in ≥ 5% of participants^f^	
COVID‐19	37 (16.2) [0.14]
Upper respiratory tract infection	24 (10.5) [0.15]
Nasopharyngitis	16 (7.0) [0.08]
Headache	14 (6.1) [0.09]
Urinary tract infection	12 (5.3) [0.06]

Abbreviations: CIDP: chronic inflammatory demyelinating polyradiculoneuropathy; COVID‐19: coronavirus disease 2019; DSMB: Data Safety Monitoring Board; ISR: injection‐site reaction; PH20: recombinant human hyaluronidase PH20; PYFU: participant‐years of follow‐up; SAE: serious adverse event; SD: standard deviation; TEAE: treatment‐emergent adverse event.

^a^ Mean (SD) study duration was 60.61 (32.87) weeks (calculated as [date of last contact − earliest date of informed consent form or date of rollover + 1 day]/ 7). ADHERE+ data cut‐off: February 16, 2024.

^b^ Event rates were calculated as the number of events divided by PYFU.

^c^ TEAEs leading to efgartigimod PH20 SC discontinuation in two or more participants were: CIDP (*n* = 5) and breast cancer (*n* = 2).

^d^ One participant had a fatal SAE of CIDP (worsening) (considered to be related to efgartigimod PH20 SC by the investigator owing to post‐exposure onset but considered not related to treatment according to the sponsor because of the long treatment duration of > 1 year with resulting clinical improvements and plausible alternative explanations). An independent DSMB reviewed the case and concluded that overall, the safety data did not reveal any concern, and one participant had a fatal SAE of cardiac arrest (considered not related to efgartigimod PH20 SC or study procedures by the investigator and sponsor).

^e^ A post hoc analysis found that ISRs were mostly mild and usually occurred within 24 h of injection; one participant (0.4%) experienced a moderate ISR; no severe ISRs were reported.

^f^ There were no recorded AEs of anaphylaxis in ADHERE or ADHERE+ at the time of data cut‐off.

SAEs were reported by 35 (15.4%; 0.25 events/PYFU) participants. The most common SAEs by preferred term were CIDP (worsening) (6/228 [2.6%]; 0.03 events/PYFU); COVID‐19 (3/228 [1.3%]; 0.01 events/PYFU); COVID‐19 pneumonia, atrial fibrillation, pneumonia, breast cancer, fall, and rib fracture (each occurred in 2/228 [0.9%]; 0.008 events/PYFU). Four (1.8%; 0.02 events/PYFU) participants had treatment‐related SAEs according to the investigator (CIDP, lymphadenitis, connective tissue disorder, type 2 diabetes, and urinary tract infection).

Two participants died during ADHERE+. One participant had a fatal SAE of CIDP (worsening) considered to be related to efgartigimod PH20 by the investigator owing to post‐exposure onset but considered not related to treatment according to the sponsor because of the long treatment duration of > 1 year with resulting clinical improvements and plausible alternative explanations. An independent data safety monitoring board reviewed the case and concluded that overall, the safety data did not reveal any concerns. Another participant (> 75 years) with a history of cardiac disease experienced cardiac complications during ADHERE+ (considered not related to efgartigimod PH20 by the investigator). The participant eventually had a fatal SAE of cardiac arrest, considered not related to efgartigimod PH20 or study procedures by the investigator and sponsor.

**FIGURE 1:**
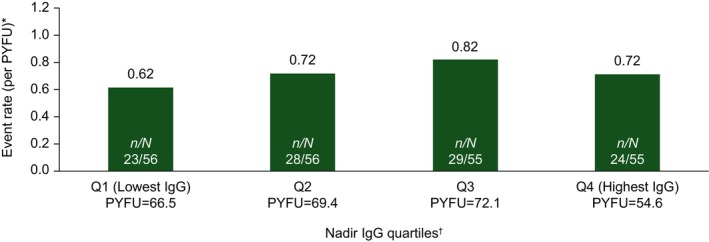
Event rate for all infections per PYFU with efgartigimod PH20 SC by lowest recorded IgG level split into quartiles in ADHERE and ADHERE+ open‐label extension study. ADHERE+ data cut‐off: February 16, 2024. *Event rates were calculated as the number of events divided by the PYFU. ^†^Concentrations of IgG were 1.260–2.370 g/L (first quartile), 2.371–3.030 g/L (second quartile), 3.031–3.900 g/L (third quartile) and 3.901–11.140 g/L (fourth quartile). IgG: immunoglobulin G; *n*: number of participants with event; *N*: total number of participants in each subgroup; PH20: recombinant human hyaluronidase PH20; PYFU: participant‐years of follow‐up; Q: quartile; SC: subcutaneous.

As efgartigimod PH20 reduces IgG levels during treatment, infections and infestations were considered as AEs of special interest. The incidence of infections (all mild to moderate in severity) with efgartigimod PH20 was low regardless of IgG level/quartile (0.62–0.82 events/PYFU across four IgG level quartiles; Figure 1). ISR event rates were low (29/228 [12.7%]; 0.27 events/PYFU), occurred more frequently in those who had received placebo versus efgartigimod PH20 during stage B (16/100 [16.0%], 0.12 events/PYFU vs. 7/99 [7.1%], 0.40 events/PYFU), and decreased with prolonged efgartigimod PH20 exposure. Among participants receiving continuous efgartigimod PH20, the incidence of ISRs was 3.0% during the first 3 months and decreased to 1.1% during the 4–6‐month period. Most ISRs were mild in severity and usually occurred within 24 h of injection; 1/228 (0.4%) participant experienced a moderate ISR and no severe (grade ≥ 3) ISRs were reported. None of the ISRs were considered serious or led to efgartigimod PH20 interruption or discontinuation, and none of the ISRs by preferred term occurred in ≥ 5% participants in the total group.

Overall, efgartigimod PH20 was considered to be well tolerated during ADHERE+ and the incidence of TEAEs did not increase with prolonged exposure to efgartigimod PH20 (participants who received efgartigimod PH20 in stage B: 2.5 events/PYFU vs. participants who received placebo during stage B: 3.5 events/PYFU).

### 3.3 Long‐Term Efficacy

**FIGURE 2:**
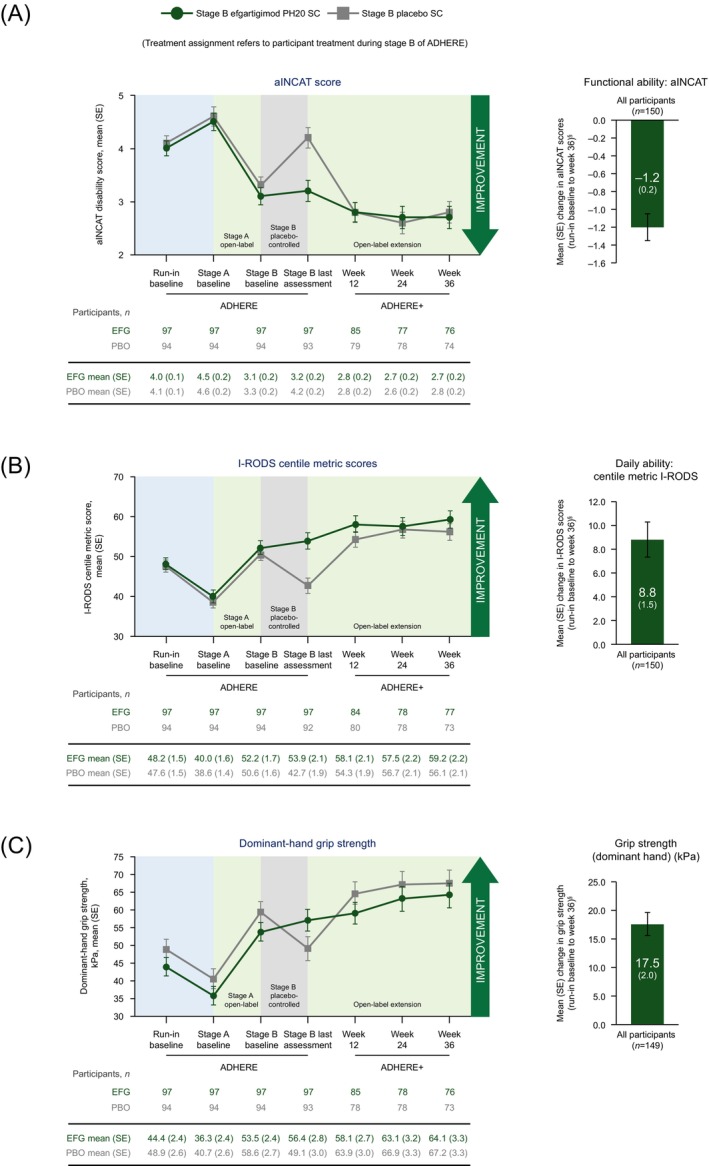
Clinically meaningful improvements among ADHERE stage A responders in the ADHERE+ open‐label extension study: (A) aINCAT score,* (B) I‐RODS centile metric scores,^†^ and (C) dominant‐hand grip strength.^‡^ ADHERE+ data cut‐off: February 16, 2024. This post hoc analysis population included efgartigimod PH20 responders in stage A with run‐in baseline values and ongoing on ADHERE+ at the time of data cut‐off (*N* = 221). Clinically meaningful improvements in mean aINCAT scores, I‐RODS scores, and dominant‐hand GS were observed in participants receiving efgartigimod PH20 continuously from run‐in baseline to ADHERE+ week 36; aINCAT scores: Mean (SE; 95% CI): −1.2 (0.22; −1.67 to −0.80); median: –1.0; I‐RODS scores: Mean (SE; 95% CI): 9.5 (2.02; 5.45 to 13.51); median: 4.0; dominant‐hand GS were observed in the continuous efgartigimod PH20 group from run‐in baseline to ADHERE+ week 36 (mean [SE; 95% CI]): 18.2 kPa (2.57 [13.11 to 23.34]; median: 12.5 kPa). *A decrease of ≥ 1 points in aINCAT score is considered a minimal clinically important difference [5, 19]. ^†^An increase of ≥ 4 points in I‐RODS score is considered a minimal clinically important difference [5, 19]. ^‡^An increase of ≥ 8 kPa in grip strength is considered a minimal clinically important difference [5, 19]. ^§^Change from run‐in baseline in ADHERE to week 36 in ADHERE+. aINCAT: adjusted Inflammatory Neuropathy Cause and Treatment; I‐RODS: Inflammatory Rasch‐built Overall Disability Scale; kPa: kilopascal; PH20: recombinant human hyaluronidase PH20; SC: subcutaneous; SE: standard error.

Participants who received efgartigimod PH20 continuously (i.e., received efgartigimod PH20 in stage B) in ADHERE had a significant reduction in the risk of relapse compared with those who received placebo in stage B [17]. A post hoc analysis was conducted to determine longitudinal improvements in mean aINCAT scores from run‐in baseline of ADHERE through week 36 of ADHERE+. In stage A responders with run‐in baseline values, clinically meaningful improvements in mean aINCAT scores were observed in participants receiving efgartigimod PH20 continuously (mean [SE; 95% confidence interval (CI)]: −1.2 [0.22; −1.67 to −0.80]; median: −1.0; *n* = 76; mean exposure duration: 85.9 weeks); in participants who received placebo during stage B, mean (standard error [SE]; 95% CI) and median changes were −1.3 (0.22; −1.69 to −0.82) and −1.0 (*n* = 74) (Figure 2A). None of the participants reported an INCAT score ≤ 1 at ADHERE run‐in baseline (*n* = 191) or stage A baseline (*n* = 220). Among participants who went through stage B and entered ADHERE+, 34.7% (68/196) of participants reached INCAT ≤ 1 at any point in ADHERE+.

Similar long‐term improvements in I‐RODS centile metric scores and dominant‐hand GS were reported from run‐in baseline to ADHERE+ week 36. Clinically meaningful improvements in mean I‐RODS scores were observed in participants receiving efgartigimod PH20 continuously from run‐in baseline to ADHERE+ week 36 (mean [SE; 95% CI]: 9.5 [2.02; 5.45 to 13.51]; median: 4.0; *n* = 77); in participants who received placebo during stage B, mean (SE; 95% CI) and median changes were 8.1 (2.12; 3.87 to 12.32) and 6.0 (*n* = 73) (Figure 2B).

Clinically meaningful improvements in mean dominant‐hand GS were observed in the continuous efgartigimod PH20 group from run‐in baseline to ADHERE+ week 36 (mean [SE; 95% CI]: 18.2 kPa [2.57; 13.11 to 23.34]; median: 12.5 kPa; *n* = 76); in participants who received placebo during stage B, mean (SE; 95% CI) and median changes were 16.7 kPa (3.16; 10.35 to 22.96) and 9.0 kPa (*n* = 73) (Figure 2C). Similar improvements were seen with non–dominant‐hand GS.

#### 3.3.1 Impact of Prior Treatment

**FIGURE 3:**
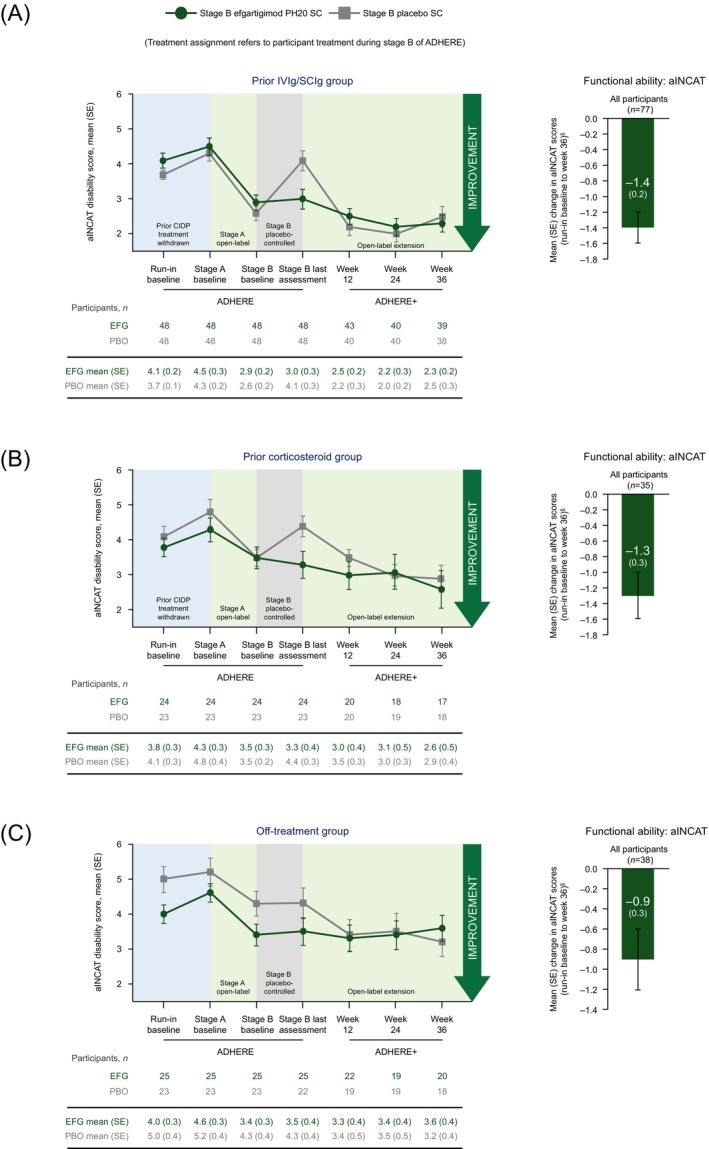
Longitudinal aINCAT from ADHERE run‐in to week 36 ADHERE+ in: (A) prior IVIg/SCIg, (B) corticosteroid and (C) off‐treatment participants. ADHERE+ data cut‐off: February 16, 2024. A decrease of ≥ 1 point in aINCAT score is considered a minimal clinically important difference [5, 19]. Off‐treatment was defined as participants who had never received CIDP treatment (CIDP treatment naïve) or who had not received CIDP treatment (corticosteroids, IVIg, or SCIg) within 6 months of study entry. Mean (SE; 95% CI) changes in aINCAT scores from run‐in baseline to ADHERE+ week 36 were −1.4 (0.2; −1.8 to −0.9) [median: −1.0], −1.3 (0.3; −1.9 to −0.7) [median: −1.0], and −0.9 (0.3; −1.5 to −0.3) [median: −1.0] for those who had received prior IVIg/SCIg, prior corticosteroids or who were off‐treatment, respectively. aINCAT: adjusted Inflammatory Neuropathy Cause and Treatment; IVIg: intravenous immunoglobulin; PH20: recombinant human hyaluronidase PH20; SC: subcutaneous; SCIg: subcutaneous immunoglobulin; SE: standard error.

Across all prior treatment subgroups, efgartigimod PH20 improved mean aINCAT scores, I‐RODS centile metric scores, and dominant‐hand GS through week 36 of ADHERE+, with improvements corresponding with time on efgartigimod PH20 treatment (Figure 3, Figures S3 and S4, Table S1). Mean (SE; 95% CI) changes in aINCAT scores from run‐in baseline to ADHERE+ week 36 were −1.4 (0.22; −1.82 to −0.93) [median: −1.0; *n* = 77], −1.3 (0.31; −1.94 to −0.69) [median: −1.0; *n* = 35] and −0.9 (0.3; −1.53 to −0.31) [median: −1.0; *n* = 38] for those who had received prior IVIg/SCIg, prior corticosteroids, or who were off‐treatment, respectively (Figure 3; Table S1).

#### 3.3.2 Disease Restabilization

Overall, disease restabilization in ADHERE+ (aINCAT scores improving to stage B baseline levels or better) occurred in the majority of participants who experienced clinical deterioration (i.e., 88 events of worsening in aINCAT scores) in stage B with continued improvements over time. Of the 90 participants who reported clinical deterioration during stage B, 88 rolled over to the open‐label extension study. Of these, one participant who had previously been treated with placebo in stage B had restabilized by ADHERE+ baseline visit. By week 4 of ADHERE+, disease restabilization was observed in 11/27 (40.7%) participants previously treated with continuous efgartigimod PH20 and in 28/51 (54.9%) of those previously treated with placebo; 10 participants had no INCAT assessment. By week 36 of ADHERE+, disease restabilization had risen to 50/70 (71.4%) participants, regardless of their stage B treatment (receiving efgartigimod PH20 continuously: *n* = 15/23; receiving placebo during stage B: *n* = 35/47); 18 participants had no INCAT assessment (Figure S5). All participants, regardless of whether they achieved disease restabilization during ADHERE+, were willing to continue efgartigimod PH20 treatment without additional rescue medication.

When efficacy outcomes were stratified by clinical deterioration status during ADHERE stage B, participants who experienced clinical deterioration during stage B demonstrated clinically meaningful improvements across all assessed outcomes from ADHERE+ baseline to week 36. Among participants who experienced clinical deterioration versus no deterioration during ADHERE stage B, mean (SE) and median changes in aINCAT score from ADHERE+ baseline to week 36 were −1.8 (0.20) and −1.0 (*n* = 70) versus −0.1 (0.10) and 0.0 (*n* = 48), respectively; I‐RODS score: 15.3 (2.21) and 9.0 (*n* = 67) versus 2.2 (0.87) and 1.0 (*n* = 48), respectively; dominant‐hand GS: 19.0 (3.05) and 12.0 (*n* = 68) versus 1.5 (1.16) and 1.0 (*n* = 48), respectively. Regardless of treatment status in stage B (i.e., efgartigimod PH20 or placebo), participants who experienced clinical deterioration in stage B demonstrated clinically meaningful improvements in efficacy outcomes (aINCAT, I‐RODS, and mean dominant‐hand GS) when continuing or re‐initiating efgartigimod PH20 treatment in ADHERE+, without requiring interventional or rescue therapy.

## 4 Discussion

Interim results from ADHERE+ indicate that long‐term (up to ~187 weeks) weekly efgartigimod PH20 SC remained well tolerated and had a similar safety profile to that seen in ADHERE [17]. Moreover, no new safety signals with efgartigimod PH20 were identified in ADHERE+ compared with the established safety profile observed in participants with myasthenia gravis in the ADAPT/ADAPT‐SC/ADAPT‐SC+ studies [20, 21], primary immune thrombocytopenia in the phase 3 randomized ADVANCE IV trial [22] and pemphigus vulgaris in a phase 2 open‐label feasibility trial [14, 23]. At the data cut‐off for this interim analysis, efgartigimod PH20 treatment was still ongoing in over 75% of participants, demonstrating favorable tolerability sustained over a prolonged treatment duration (mean [SD], 58.0 [33.8] weeks).

Consistent with findings from the ADHERE study, FcRn blockade with efgartigimod PH20 also led to clinically meaningful improvements in ADHERE+. Clinically meaningful improvements were reported in mean aINCAT (decreased by 1.2 points), I‐RODS (increase of 8.8 points), and GS scores (increase of 17.5 kPa) from ADHERE run‐in baseline (prior CIDP treatments discontinued) to ADHERE+ week 36; these improvements coincided with time on efgartigimod PH20 treatment. Notably, ADHERE stage A responders who received placebo during stage B and experienced worsening subsequently demonstrated improvements with efgartigimod PH20 during ADHERE+. Long‐term clinical efficacy of efgartigimod PH20 during ADHERE+ was demonstrated across assessments regardless of prior treatment status, with greater improvements seen over time on efgartigimod PH20 treatment. When outcomes were evaluated by prior treatment status, stage A responders previously treated with IVIg/SCIg reported clinically meaningful improvements over time on efgartigimod PH20, suggesting improved efficacy relative to their prior IVIg/SCIg treatment; these participants demonstrated the greatest aINCAT improvement (mean aINCAT scores decreased by 1.4 points from run‐in baseline) by ADHERE+ week 36.

Among the 88 participants with clinical deterioration (i.e., relapse) who rolled over to ADHERE+, one participant (placebo group) had restabilized by ADHERE+ baseline. Disease restabilization occurred within 4 weeks of ADHERE+ (i.e., first post‐baseline visit) in 50% (39/78) of assessed participants. By ADHERE+ week 36, this had risen to over 70% (50/70) of participants, with restabilization in nearly three‐quarters of those who had received placebo in stage B and two‐thirds of those who had received efgartigimod PH20 continuously. Notably, restabilization was observed in participants without the need for interventional or rescue therapy.

No new safety or tolerability signals were reported with efgartigimod PH20 during ADHERE+ through week 36. Given the long duration of this open‐label extension study, accumulation of TEAEs over time was anticipated (3.1 events/PYFU). Most common TEAEs (≥ 5% participants) included COVID‐19, upper respiratory tract infection, nasopharyngitis, headache, and urinary tract infection, each with low event rates (≤ 0.15 events/PYFU). Incidence of SAEs in ADHERE+ was low (15.4%) and comparable with rates observed in long‐term IVIg treatment during the ICE study, in which 12.2% of participants reported an SAE [24]. Some cases of the SAE of CIDP (worsening) were reported during ADHERE+; however, such occurrences appeared to be sporadic with a minimal event rate (*n* = 6/228; 0.03 events/PYFU), and in line with expectations from prior treatment switches/regimens and the relapsing nature of the disease [4]. Notably, discontinuations due to CIDP (worsening) were largely deemed unrelated to treatment. Furthermore, such discontinuations were reported in 2% (5/228) of participants, reflecting a low incidence compared with relapse frequencies previously reported in open‐label extension trials in CIDP [25]. All participants who discontinued ADHERE+, irrespective of the reason, were managed per the local standard of care as determined by the treating physician.

Two fatalities were reported during ADHERE+: a participant > 75 years of age with a history of cardiac disease experienced multiple serious cardiac complications (considered not related to efgartigimod PH20 by the investigator) leading up to a fatal SAE of cardiac arrest. The events were assessed by the investigator and sponsor and considered not related to efgartigimod PH20 or study procedures. Another participant had a fatal SAE of CIDP (worsening). Although the investigator considered the event to possibly be related to efgartigimod PH20 based on timing, the sponsor assessed the event as unrelated to treatment given the long treatment duration with efgartigimod PH20, clinical improvements, and alternative explanations. Furthermore, no safety concerns were identified upon review of the case by an independent data safety monitoring board.

ISRs were mostly mild and usually occurred within 24 h of injection; the incidence of ISRs were higher during the first 3 months and decreased over time. Mitigation strategies for ISRs (e.g., optimization of injection angle, needle gauge, and bevel design) may be considered to help reduce their occurrence [26, 27]. In contrast to agents that can increase infection risk (e.g., immunosuppressants, immunomodulatory agents) [27, 28], efgartigimod PH20 was associated with a low incidence of infection, all mild to moderate in severity, regardless of IgG level/quartile (0.62–0.82 events/PYFU across four IgG level quartiles). Indeed, efgartigimod selectively reduces IgG without impacting antibody production or other parts of the immune system [9, 10, 12], and low rates of infection were seen regardless of IgG level. Thus, the safety profile of long‐term efgartigimod PH20 treatment was in line with approved regimens for CIDP [24].

Overall, the different baseline definitions used for participants who received efgartigimod PH20 versus placebo at ADHERE stage B and application of distinct baselines depending on the objective of each analysis allowed for critical assessment of efgartigimod PH20 treatment outcomes across the different stages of ADHERE–ADHERE+. Potential limitations of ADHERE+ included the open‐label design of the study, which may lead to participant and/or investigator bias, as well as the exploratory nature of the post hoc analyses.

In conclusion, this interim analysis from ADHERE+ shows that prolonged efgartigimod PH20 treatment in participants with CIDP was well tolerated, with continuous or sustained improvements in clinical assessments of functional ability, daily activity, and strength.

## Author Contributions

**Jeffrey A. Allen** and **Richard A. Lewis:** conceptualization (lead). **Geoffrey Istas**, **Satoshi Kuwabara**, **Trevor Mole**, **Luis Querol**, **Simon Rinaldi**, **Mark Stettner**, **Benjamin Van Hoorick:** conceptualization. **Trevor Mole** and **Geoffrey Istas:** data curation. **Trevor Mole:** formal analysis. **Jeffrey A. Allen**, **Richard A. Lewis**, **Satoshi Kuwabara**, **Luis Querol**, **Simon Rinaldi**, **Mark Stettner**, **Benjamin Van Hoorick:** investigation. **Jeffrey A. Allen**, **Richard A. Lewis**, **Satoshi Kuwabara**, **Luis Querol**, **Simon Rinaldi**, **Mark Stettner**, **Benjamin Van Hoorick:** methodology. **Geoffrey Istas:** project administration. **Geoffrey Istas**, **Benjamin Van Hoorick:** supervision. **Geoffrey Istas**, **Jeffrey A. Allen**, **Trevor Mole**, **Benjamin Van Hoorick:** validation. **Jeffrey A. Allen**, **Richard A. Lewis:** writing – original draft (lead). **Geoffrey Istas**, **Satoshi Kuwabara**, **Trevor Mole**, **Luis Querol**, **Simon Rinaldi**, **Mark Stettner**, **Benjamin Van Hoorick:** writing – original draft. **Jeffrey A. Allen** and **Richard A. Lewis:** writing – review and editing (lead). **Geoffrey Istas**, **Satoshi Kuwabara**, **Trevor Mole**, **Luis Querol**, **Simon Rinaldi**, **Mark Stettner**, **Benjamin Van Hoorick:** writing – review and editing.

## Funding

This study was funded by argenx. The funders designed the trial; collected, analyzed, and interpreted the data; and supported the manuscript development in collaboration with the authors.

## Ethics Statement

ADHERE+ was conducted in accordance with the protocol and consensus ethical principles derived from international guidelines (i.e., the Declaration of Helsinki, Council for International Organizations of Medical Sciences International Ethical Guidelines, applicable International Council for Harmonization of Technical Requirements for Pharmaceuticals for Human Use Good Clinical Practice Guidelines) and other applicable laws and regulations.

## Conflicts of Interest

J.A.A. has received consultancy fees from Akcea, Alexion, Alnylam, Annexon, argenx, CSL Behring, Grifols, Immunovant, ImmuPharma, Johnson & Johnson, Pfizer, and Takeda. M.S. has served on the scientific advisory boards and/or steering committee member and/or received speaker honoraria, travel funding, or honoraria for medical writing from argenx, Bayer, Biogen, Biotest, CSL Behring, Genzyme, Grifols, Immunovant, Kedrion, Merck, Novartis, Octapharma, PPTA, Roche, Sanofi‐Aventis, TEVA, and UCB. G.I., T.M., and B.V.H. are employees of argenx. S.K. has received payment or honoraria for lectures or presentations from Alexion, argenx, CSL Behring, and Takeda. L.Q. has received research grants from CIBERER, Fundació La Marató, GBS‐CIDP Foundation International, Grifols, Instituto de Salud Carlos III—Ministry of Economy and Innovation (Spain), and UCB; received speaker or expert testimony honoraria from Annexon Biosciences, Alnylam Pharmaceuticals, argenx, Avilar Therapeutics, Biogen, CSL Behring, Dianthus, Janssen, LFB, Lundbeck, Merck, Novartis, Octapharma, Roche, Sanofi, and UCB; is a steering committee member for Sanofi; and is a Principal Investigator for UCB. S.R. has received consultancy fees from Annexon, argenx, the Beijing Association of Holistic and Integrated Medicine, British Medical Association, CSL Behring, Dianthus, Excemed, Fresenius, GBS/CIDP Foundation International, Guillain‐Barré syndrome and Related Inflammatory Neuropathies (GAIN) charity, Hansa Biopharma, the Irish Institute of Clinical Neuroscience, Medical Research Council (UK), National Institute of Health Research (NIHR), the Pathological Society of Great Britain Ireland, Peripheral Nerve Society, Takeda, UCB, the University of Oxford's John Fell Fund, and Wellcome Trust. R.A.L. has received consultancy fees from Akcea, Alnylam, Amgen, argenx, Biotest, Cartesian, CSL Behring, Dianthus, Grifols, Immunovant, Intellia, Nervosave, Nuvig, Sanofi, Takeda, and TG Therapeutics.

## Supporting information


**Table S1:** Longitudinal changes in efficacy outcomes scores from ADHERE run‐in to week 36 ADHERE+ in prior IVIg/SCIg, corticosteroid, and off‐treatment participants.
**Figure S1:** The ADHERE and ADHERE+ open‐label extension study designs.
**Figure S2:** Participant treatment status in the ADHERE+ open‐label extension study.
**Figure S3:** Longitudinal I‐RODS centile metric scores from ADHERE run‐in to week 36 ADHERE+ in: (A) prior IVIg/SCIg, (B) corticosteroid, and (C) off‐treatment participants.
**Figure S4:** Longitudinal dominant‐hand grip strength scores from ADHERE run‐in to week 36 ADHERE+ in: (A) prior IVIg/SCIg, (B) corticosteroid, and (C) off‐treatment participants.
**Figure S5:** Participants with disease restabilization* in the ADHERE+ open‐label extension study.

## Data Availability

argenx is committed to responsible data sharing regarding the clinical trials it funds. Included in this commitment is access to anonymized individual‐level and trial‐level data (analysis data sets), and other information (e.g., protocols and clinical study reports), as long as the trial is not part of an ongoing or planned regulatory submission. These clinical trial data can be requested by qualified researchers who engage in rigorous independent scientific research and will only be provided after review and approval of a research proposal and statistical analysis plan and execution of a data sharing agreement. Data requests can be submitted at any time, and the data will be accessible for 12 months. Requests can be submitted to esr@argenx.com.
