# Supplementary material for: Long‐Term Safety and Efficacy of Efgartigimod PH20 in Chronic Inflammatory Demyelinating Polyradiculoneuropathy: ADHERE/ADHERE+ Trial Interim Analysis
Source: J Peripher Nerv Syst. 2026 Jul 16;31(3):e70140. doi: 10.1111/jns.70140 (PMC13375598; doi:10.1111/jns.70140)
Supplement: Supplementary file 1 — Table S1: Longitudinal changes in efficacy outcomes scores from ADHERE run‐in to week 36 ADHERE+ in prior IVIg/SCIg, corticosteroid, and off‐treatment participants. Figure S1: The ADHERE and ADHERE+ open‐label extension study designs. Figure S2: Participant treatment status in the ADHERE+ open‐label extension study. Figure S3: Longitudinal I‐RODS centile metric scores from ADHERE run‐in to week 36 ADHERE+ in: (A) prior IVIg/SCIg, (B) corticosteroid, and (C) off‐treatment participants. Figure S4: Longitudinal dominant‐hand grip strength scores from ADHERE run‐in to week 36 ADHERE+ in: (A) prior IVIg/SCIg, (B) corticosteroid, and (C) off‐treatment participants. Figure S5: Participants with disease restabilization* in the ADHERE+ open‐label extension study. [file JNS-31-0-s001.docx]

**Supplemental material**

**Long-Term Safety and Efficacy of Efgartigimod PH20 in Chronic Inflammatory Demyelinating Polyradiculoneuropathy: ADHERE/ADHERE+ Trial Interim Analysis**

Allen JA, et al.

**SUPPLEMENTAL TABLE 1 |** Longitudinal changes in efficacy outcomes scores from ADHERE run-in to week 36 ADHERE+ in prior IVIg/SCIg, corticosteroid, and off-treatment participants.

1. **aINCAT Scores**

|  | **IVIg/SCIg group** | | | **Corticosteroid group** | | | **Off-treatment group** | | |  |
| --- | --- | --- | --- | --- | --- | --- | --- | --- | --- | --- |
|  | **Actual values,  mean (95% CI), median** | | | **Actual values,  mean (95% CI), median** | | | **Actual values,  mean (95% CI), median** | | |  |
|  | **Efgartigimod  PH20 SC  (*N*=48)** | **Placebo**  **(*N*=48)** | **All participants**  **(*N*=96)** | **Efgartigimod  PH20 SC  (*N*=24)** | **Placebo**  **(*N*=23)** | **All participants**  **(*N*=47)** | **Efgartigimod  PH20 SC  (*N*=39)** | **Placebo**  **(*N*=39)** | **All  participants**  **(*N*=78)** |  |
| Run-in baseline | 4.1  (3.6, 4.5),  4.0 | 3.7  (3.4, 4.0),  4.0 | 3.9  (3.6, 4.1),  4.0 | 3.8  (3.2, 4.3),  3.0 | 4.1  (3.5, 4.8),  4.0 | 3.9  (3.5, 4.4),  3.0 | 4.0  (3.5, 4.6),  4.0 | 5.0  (4.2, 5.8), 5.0 | 4.5  (4.0, 5.0),  4.0 |  |
| Stage A baseline | 4.5  (4.0, 5.1),  4.0 | 4.3  (3.9, 4.7),  4.0 | 4.4  (4.1, 4.7),  4.0 | 4.3  (3.6, 5.0),  4.0 | 4.8  (4.0, 5.6),  4.0 | 4.5  (4.0, 5.0),  4.0 | 4.6  (4.0, 5.1),  4.0 | 5.2  (4.4, 6.1), 6.0 | 4.9  (4.4, 5.4),  4.0 |  |
| Stage B baseline | 2.9 (2.4, 3.3),  3.0 | 2.6  (2.2, 3.1),  3.0 | 2.8  (2.5, 3.1),  3.0 | 3.5  (2.8, 4.1),  3.0 | 3.5  (3.0, 4.0),  3.0 | 3.5  (3.1, 3.9),  3.0 | 3.4  (2.8, 4.0),  3.0 | 4.3 (3.6, 5.0), 4.0 | 3.8  (3.4, 4.3),  4.0 |  |
| Stage B last assessment | 3.0  (2.4, 3.6),  3.0 | 4.1  (3.6, 4.7),  4.0 | 3.6  (3.1, 4.0),  3.0 | 3.3 (2.5, 4.1),  3.0 | 4.4  (3.8, 5.1), 5.0 | 3.8  (3.3, 4.4),  4.0 | 3.5  (2.7, 4.3),  4.0 | 4.3  (3.4, 5.2), 4.0 | 3.9  (3.3, 4.5),  4.0 |  |
| ADHERE+ week 36 | 2.3  (1.8, 2.8),  3.0 | 2.5  (1.9, 3.1),  3.0 | 2.4  (2.0, 2.8),  3.0 | 2.6  (1.4, 3.7),  3.0 | 2.9  (2.1, 3.7),  3.0 | 2.7  (2.1, 3.4),  3.0 | 3.6  (2.8, 4.4),  4.0 | 3.2  (2.3, 4.1), 3.0 | 3.4  (2.9, 4.0),  3.0 |  |

1. **I-RODS Centile Metric Scores**

|  | **IVIg/SCIg group** | | | | **Corticosteroid group** | | | | **Off-treatment group** | | |  |
| --- | --- | --- | --- | --- | --- | --- | --- | --- | --- | --- | --- | --- |
|  | **Actual values,  mean (95% CI), median** | | | | **Actual values,  mean (95% CI), median** | | | | **Actual values,  mean (95% CI), median** | | |  |
|  | **Efgartigimod  PH20 SC  (*N*=48)** | **Placebo**  **(*N*=48)** | **All participants**  **(*N*=96)** | **Efgartigimod  PH20 SC  (*N*=24)** | | **Placebo**  **(*N*=23)** | **All participants**  **(*N*=47)** | **Efgartigimod  PH20 SC  (*N*=39)** | | **Placebo**  **(*N*=39)** | **All  participants**  **(*N*=78)** |  |
| Run-in baseline | 49.2  (45.0, 53.3), 48.5 | 50.8  (46.9, 54.7), 49.0 | 50.0  (47.2, 52.8), 49.0 | 52.5  (45.6, 59.4),  53.0 | | 46.9  (39.7, 54.1),  46.0 | 49.7  (44.9, 54.6),  48.0 | 42.2  (37.9; 46.5), 41.0 | | 41.6  (35.4; 47.9), 40.0 | 41.9  (38.3; 45.5), 40.5 |  |
| Stage A baseline | 40.5  (35.7, 45.3), 41.0 | 41.2  (36.9, 45.4), 40.5 | 40.8  (37.7, 44.0), 41.0 | 44.7  (37.2, 52.1), 41.0 | | 37.3  (32.3, 42.3), 37.0 | 41.0  (36.6, 45.5), 40.0 | 34.8  (31.5; 38.1), 35.0 | | 34.4  (28.6; 40.3), 34.0 | 34.6  (31.5; 37.8), 34.5 |  |
| Stage B baseline | 55.8  (50.7, 60.8), 54.0 | 57.7  (53.0, 62.5), 57.5 | 56.7  (53.4, 60.1), 54.5 | 52.4  (45.0, 59.7), 51.5 | | 45.5  (41.4, 49.5), 43.0 | 49.0  (44.8, 53.2), 48.0 | 45.0  (39.0; 51.1), 41.0 | | 40.9  (35.9; 45.9) 42.0 | 43.0  (39.2; 46.9), 41.5 |  |
| Stage B last assessment | 58.7  (52.6, 64.8), 57.0 | 45.0  (38.6, 51.4), 43.0 | 51.9  (47.4, 56.5), 50.0 | 50.4  (42.8, 58.0),  52.5 | | 38.5  (34.5, 42.4), 37.0 | 44.6  (40.0, 49.1), 42.0 | 48.2  (40.3; 56.1), 40.0 | | 42.2  (34.6; 49.9), 37.0 | 45.4  (40.0; 50.8), 39.0 |  |
| ADHERE+ week 36 | 63.2(56.9, 69.5) 64.0 | 62.8  (56.3, 69.3) 63.0 | 63.0  (58.6, 67.5) 63.0 | 57.6  (48.4, 66.8), 57.0 | | 47.9  (42.8, 53.0),  45.5 | 52.6  (47.4, 57.8),  52.0 | 52.7  (44.1; 61.3), 51.0 | | 50.5  (42.5; 58.5), 50.5 | 51.7  (46.0; 57.3), 51.0 |  |

1. **Dominant-Hand Grip Strength**

|  | **IVIg/SCIg group** | | | | **Corticosteroid group** | | | | **Off-treatment group** | | |  |
| --- | --- | --- | --- | --- | --- | --- | --- | --- | --- | --- | --- | --- |
|  | **Actual values,  mean (95% CI), median, kPa** | | | | **Actual values,  mean (95% CI), kPa** | | | | **Actual values,  mean (95% CI), kPa** | | |  |
|  | **Efgartigimod  PH20 SC  (*N*=48)** | **Placebo**  **(*N*=48)** | **All participants**  **(*N*=96)** | **Efgartigimod  PH20 SC  (*N*=24)** | | **Placebo**  **(*N*=23)** | **All participants**  **(*N*=47)** | **Efgartigimod  PH20 SC  (*N*=39)** | | **Placebo**  **(*N*=39)** | **All  participants**  **(*N*=78)** |  |
| Run-in baseline | 42.1  (35.3, 48.9), 47.5 | 50.2  (43.3, 57.2), 52.0 | 46.2  (41.3, 51.0), 50.0 | 50.6  (39.0, 62.2), 46.5 | | 51.8  (41.6, 62.1), 49.0 | 51.2  (43.7, 58.7), 48.0 | 42.9  (35.1, 50.8), 45.0 | | 43.1  (31.0, 55.2), 39.0 | 43.0  (36.2, 49.8), 41.5 |  |
| Stage A baseline | 34.8  (28.0, 41.5), 36.5 | 39.8  (33.1, 46.5), 42.5 | 37.3  (32.6, 42.0), 37.5 | 40.8  (28.9, 52.7), 39.5 | | 45.8  (34.7, 56.9), 42.0 | 43.3  (35.4, 51.1), 41.0 | 34.9  (27.4, 42.4), 37.0 | | 37.4  (25.5, 49.4), 38.0 | 36.1  (29.5, 42.8), 37.5 |  |
| Stage B baseline | 52.8  (46.4, 59.1), 51.5 | 61.7  (54.5, 68.9), 59.0 | 57.2  (52.4, 62.0), 54.0 | 56.1  (43.6, 68.7), 57.0 | | 60.6  (49.9, 71.2), 57.0 | 58.3  (50.3, 66.3), 57.0 | 52.3  (44.2, 60.4), 57.0 | | 50.0  (37.0, 63.1), 50.0 | 51.2  (44.0, 58.5), 52.5 |  |
| Stage B last assessment | 56.2  (48.8, 63.5), 54.0 | 48.4  (39.3, 57.5), 46.0 | 52.3  (46.5, 58.1), 49.0 | 56.5  (43.2, 69.9), 50.5 | | 51.0  (38.7, 63.4), 49.0 | 53.9  (45.1, 62.7), 50.0 | 56.6  (45.5, 67.8), 53.0 | | 48.5  (38.1, 58.9), 48.5 | 52.8  (45.3, 60.3), 51.0 |  |
| ADHERE+ week 36 | 64.4  (56.4, 72.4), 65.0 | 66.1  (56.2, 76.0), 62.0 | 65.2  (59.1, 71.4), 63.5 | 68.4  (48.2, 88.5), 74.0 | | 72.2  (57.6, 86.8), 66.0 | 70.3  (58.7, 82.0), 67.0 | 59.7  (48.1, 71.3), 59.0 | | 64.3  (51.5, 77.1), 60.5 | 61.9  (53.6, 70.1), 60.0 |  |

ADHERE+ data cut-off: February 16, 2024.
Off-treatment was defined as participants who had never received CIDP treatment (CIDP treatment naïve) or who had not received CIDP treatment (corticosteroids, IVIg, or SCIg) within 6 months of study entry.

Abbreviations: aINCAT: adjusted Inflammatory Neuropathy Cause and Treatment; IVIg: intravenous immunoglobulin; I-RODS: Inflammatory Rasch-built Overall Disability Scale; kPa: kilopascal; PH20: recombinant human hyaluronidase PH20; SC: subcutaneous; SCIg: subcutaneous immunoglobulin.

**SUPPLEMENTAL FIGURE 1 |** The ADHERE [16] and ADHERE+ open-label extension study designs.


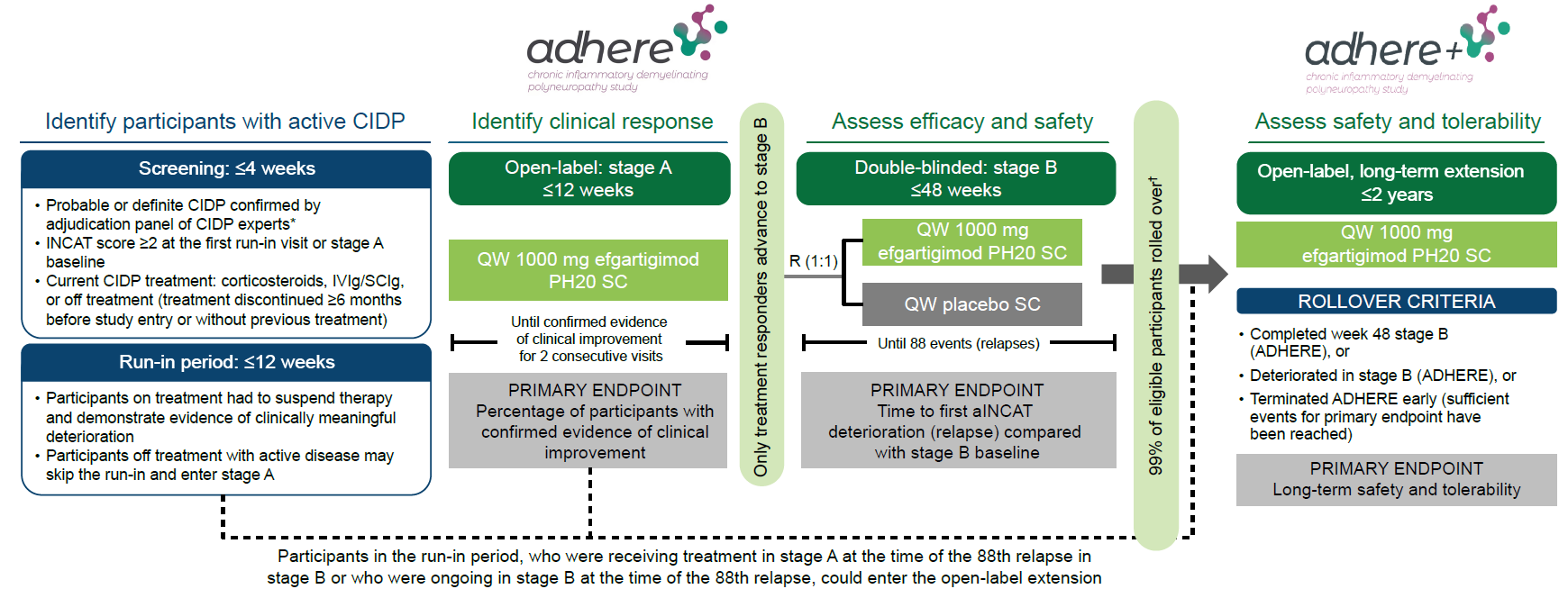


ECI was defined as a clinical improvement on the parameters in which the participant worsened during run-in (≥4-point increase in I-RODS and/or ≥8-kPa increase in mean grip strength) or clinical improvement (≥1-point decrease) in INCAT. ECI was confirmed after these criteria were met after 4 injections and 2 consecutive visits.

*According to 2010 criteria of the European Federation of Neurological Societies/Peripheral Nerve Society [17], progressing or relapsing forms.
^†^*n*=228/229. 229 participants enrolled in ADHERE+, including 3 participants who inadvertently rolled over without meeting per-protocol inclusion criteria. The safety population for ADHERE+ included 228 participants who received at least one dose of efgartigimod PH20 SC in the open-label extension period, as one participant discontinued before receiving the first dose of efgartigimod PH20 SC.
Abbreviations: aINCAT: adjusted Inflammatory Neuropathy Cause and Treatment; CIDP: chronic inflammatory demyelinating polyradiculoneuropathy; ECI: evidence of clinical improvement; INCAT: Inflammatory Neuropathy Cause and Treatment; IVIg: intravenous immunoglobulin; PH20: recombinant human hyaluronidase PH20; QW: once weekly; R: randomization; SC: subcutaneous; SCIg: subcutaneous immunoglobulin.

**SUPPLEMENTAL FIGURE 2 |** Participant treatment status in the ADHERE+ open-label extension study.


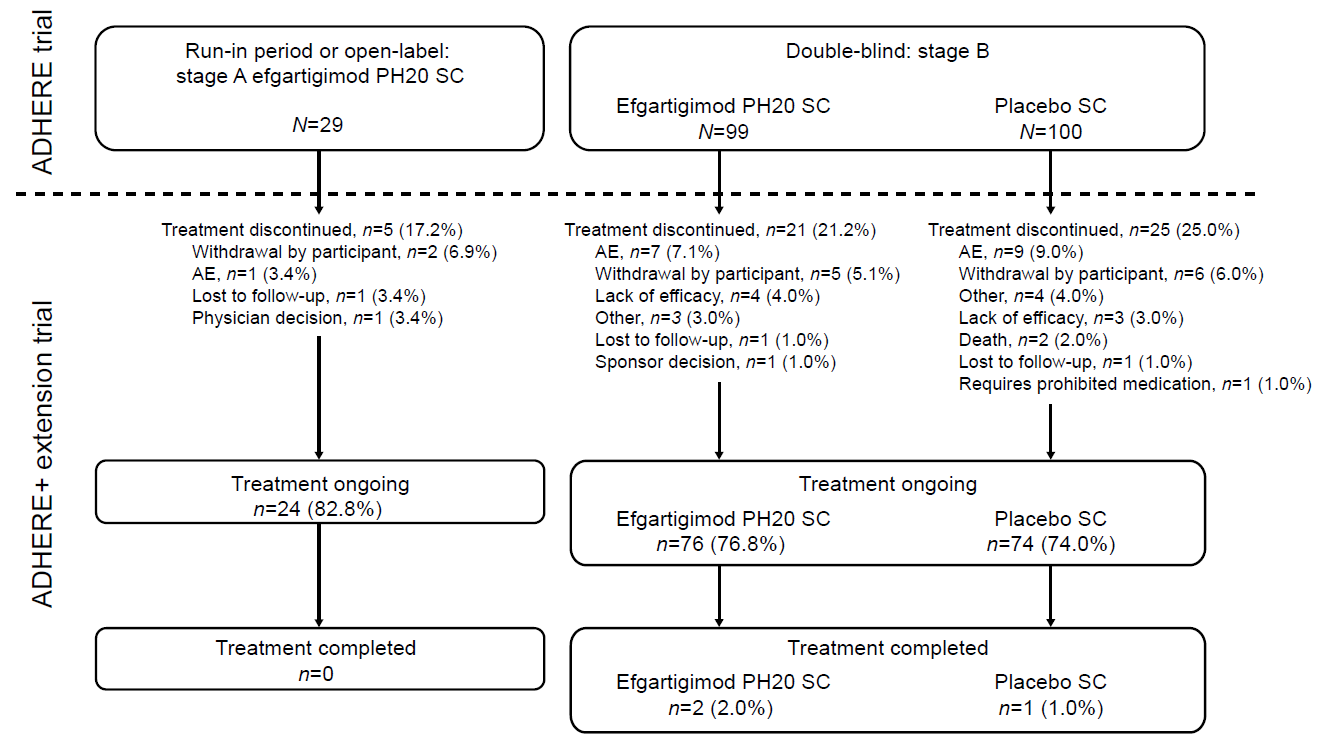


One participant receiving placebo in stage B withdrew from ADHERE+ before receiving efgartigimod PH20.

*Apart from the 9 participants who discontinued treatment due to an AE, one additional participant discontinued treatment due to an AE. However, as this participant died during the study, the reason for discontinuation was documented as “death”.

ADHERE+ was planned in 48-week treatment periods, and participants could complete the study after 1 or more 48-week treatment periods.

Abbreviations: AE: adverse event; PH20: recombinant human hyaluronidase PH20; SC: subcutaneous.

**SUPPLEMENTAL FIGURE 3 |** Longitudinal I-RODS centile metric scores from ADHERE run-in to week 36 ADHERE+ in: (A) prior IVIg/SCIg, (B) corticosteroid, and (C) off-treatment participants.

**
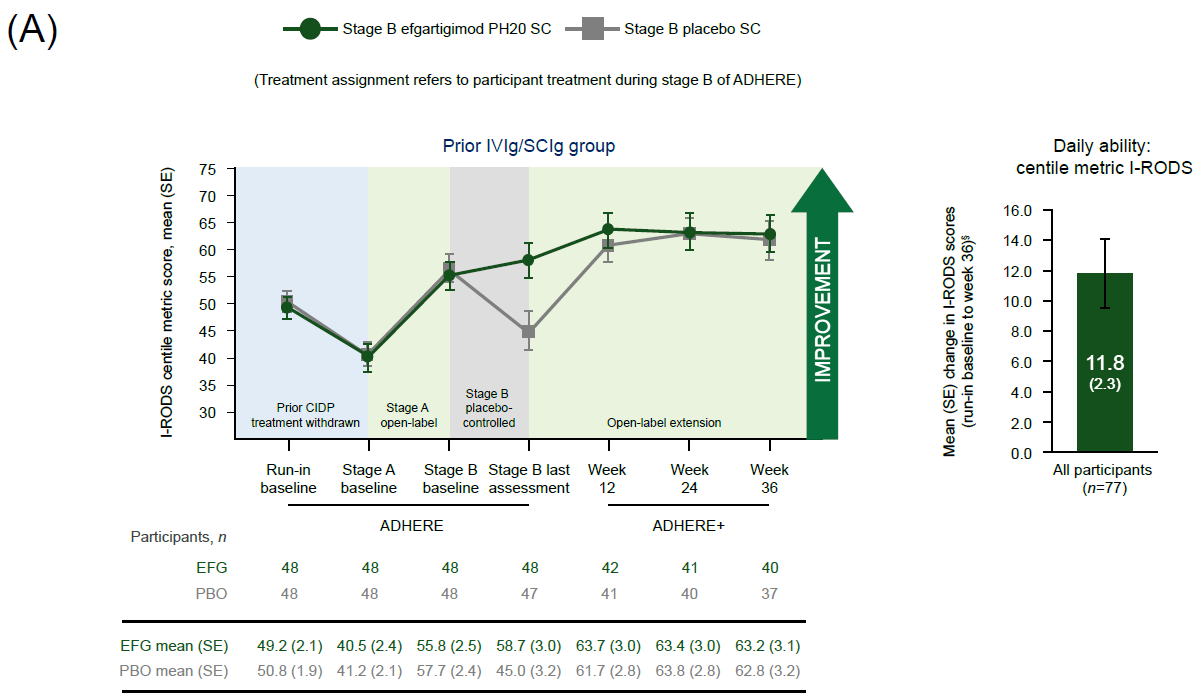
**

**
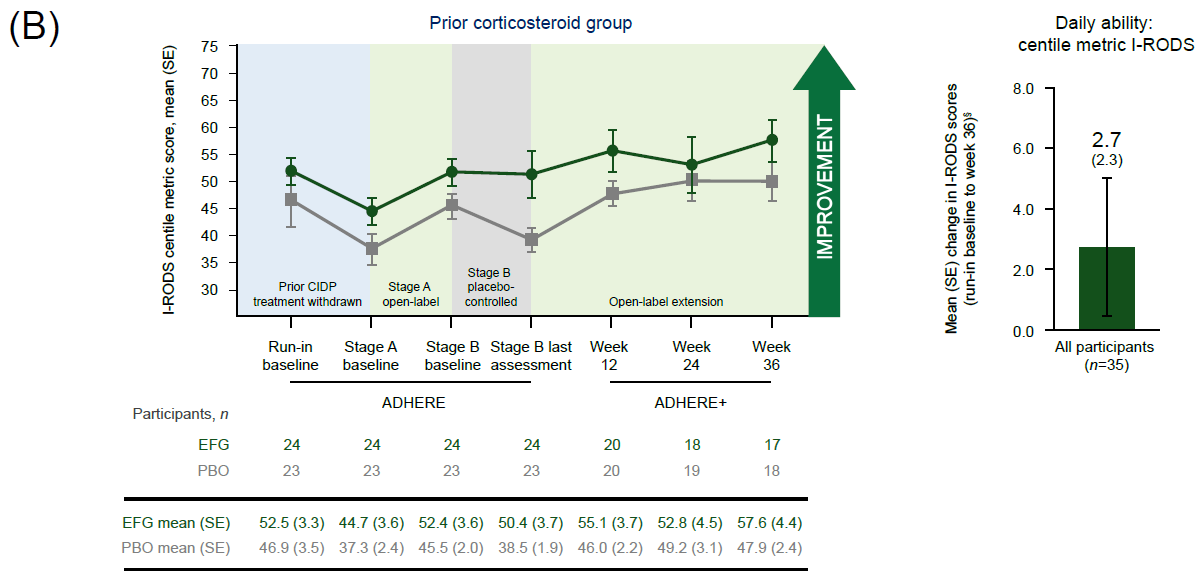
**

**
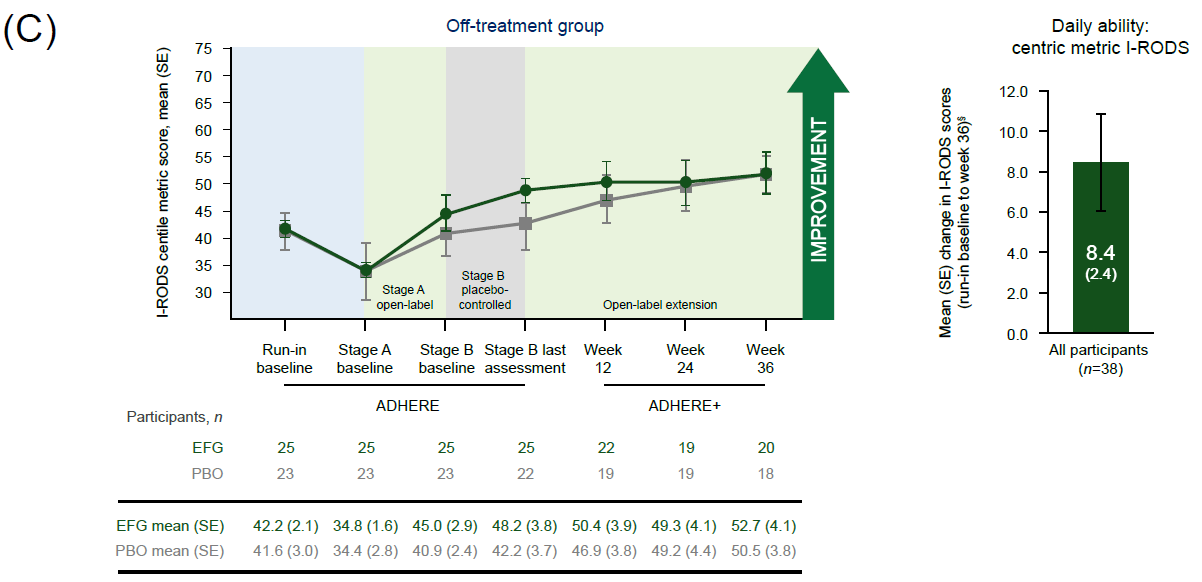
**

ADHERE+ data cut-off: February 16, 2024.
An increase of ≥4 points in I-RODS score is considered a minimal clinically important difference [5,18].

Off-treatment was defined as participants who had never received CIDP treatment (CIDP treatment naïve) or who had not received CIDP treatment (corticosteroids, IVIg or SCIg) within 6 months of study entry.

Mean (SE; 95% CI) changes in I-RODS scores from run-in baseline to ADHERE+ week 36 were 11.8 (2.3; 7.2 to 16.4) [median: 8.0], 2.7 (2.3; –1.9 to 7.4) [median: 0.0] and 8.4 (2.4; 3.5 to 13.4) [median: 5.0] for those who had received prior IVIg/SCIg, prior corticosteroids, or who were off-treatment, respectively.

Abbreviations: I-RODS: Inflammatory Rasch-built Overall Disability Scale; IVIg: intravenous immunoglobulin; PH20: recombinant human hyaluronidase PH20; SC: subcutaneous; SCIg: subcutaneous immunoglobulin; SE: standard error.

**SUPPLEMENTAL FIGURE 4 |** Longitudinal dominant-hand grip strength scores from ADHERE run-in to week 36 ADHERE+ in: (A) prior IVIg/SCIg, (B) corticosteroid, and (C) off-treatment participants.

**
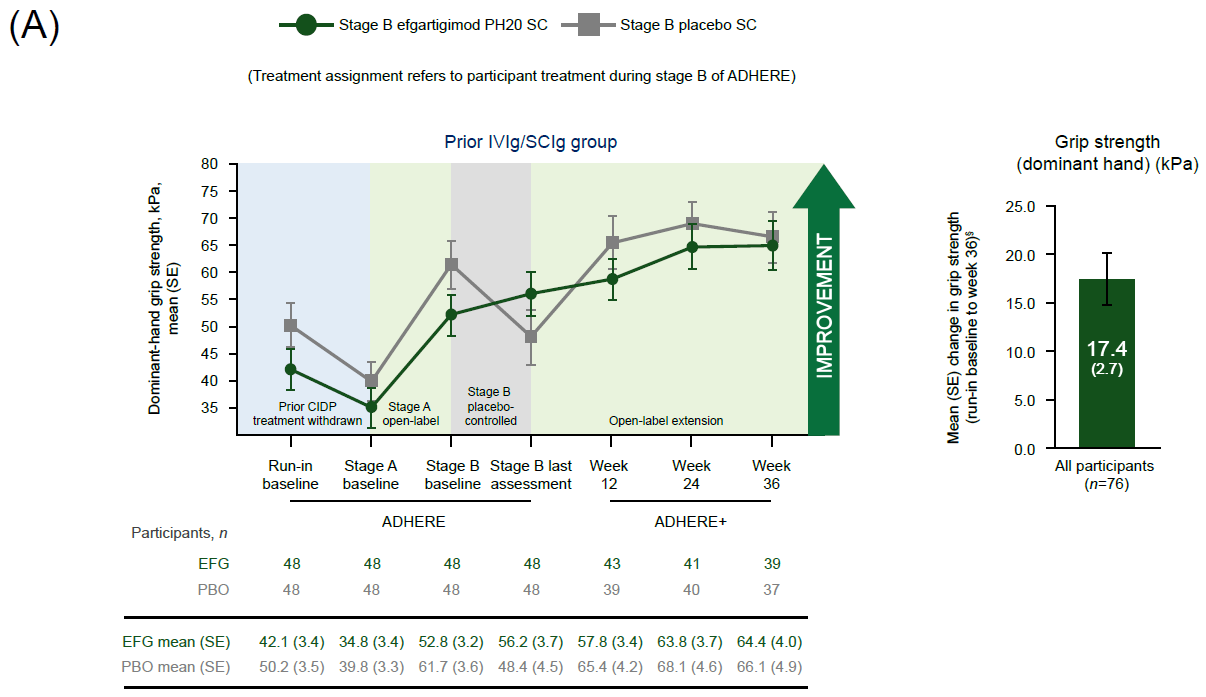
**

**
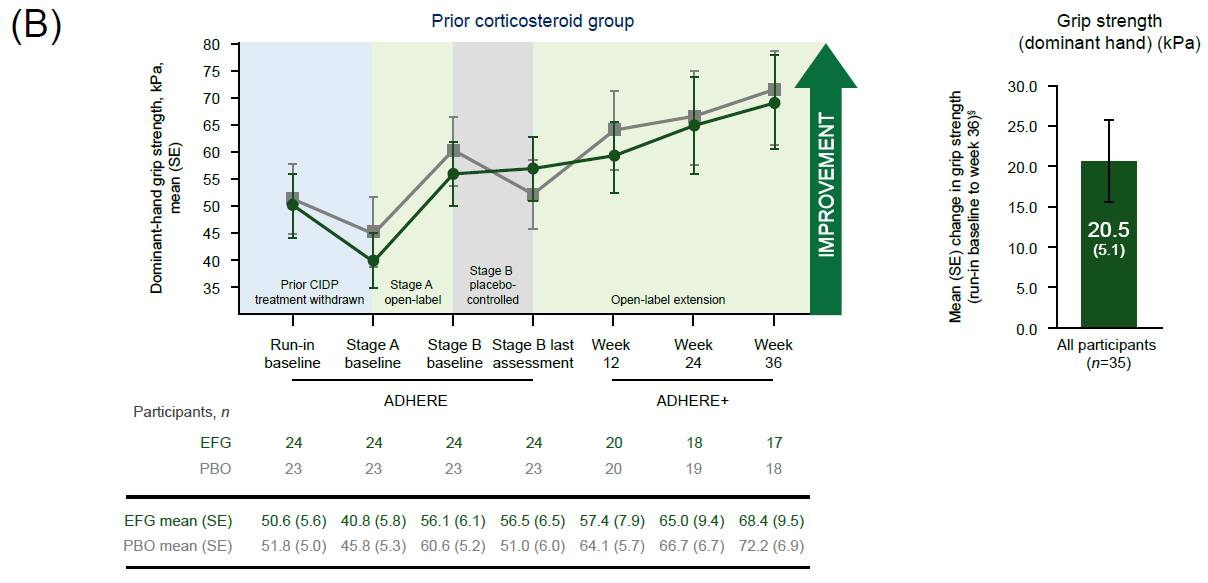
**


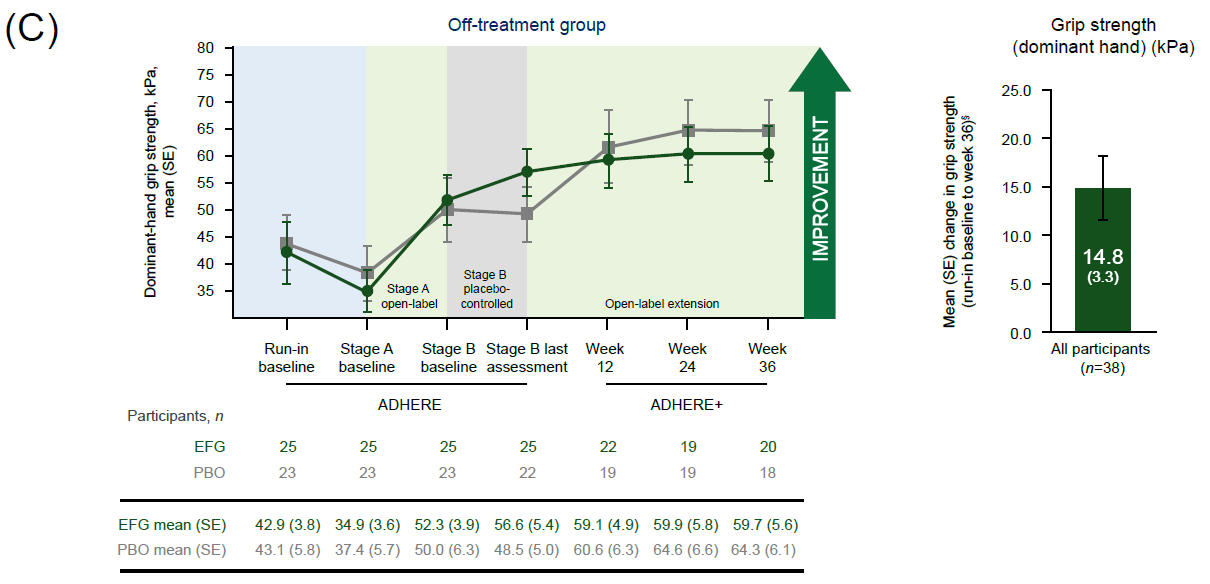


ADHERE+ data cut-off: February 16, 2024.
An increase of ≥8 kPa in grip strength is considered a minimal clinically important difference [5,18].
Mean (SE; 95% CI) changes in dominant-hand grip strength scores from run-in baseline to ADHERE+ week 36 were 17.4 (2.7; 11.9 to 22.8) [median: 12.0], 20.5 (5.1; 10.1 to 31.0) [median: 10.0] and 14.8 (3.3; 8.1 to 21.5) [median: 8.5] for those who had received prior IVIg/SCIg, prior corticosteroids, or who were off-treatment, respectively.

Abbreviations: IVIg: intravenous immunoglobulin; kPa: kilopascal; PH20: recombinant human hyaluronidase PH20; SC: subcutaneous; SCIg: subcutaneous immunoglobulin; SE: standard error.

**SUPPLEMENTAL FIGURE 5 |** Participants with disease restabilization* in the ADHERE+ open-label extension study.


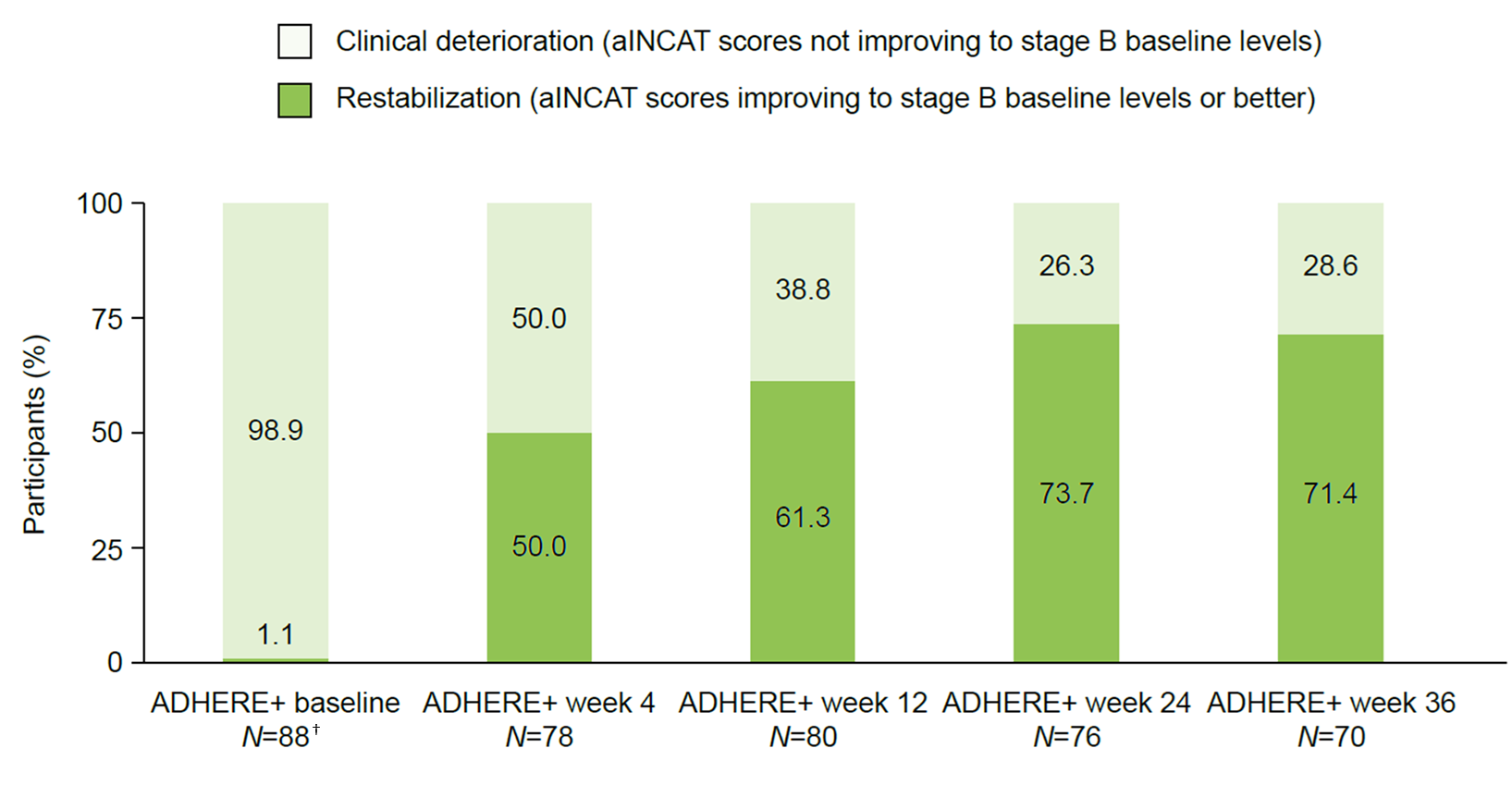
ADHERE+ data cut-off: February 16, 2024.
This post-hoc analysis included participants in ADHERE+ with disease relapse in ADHERE stage B. Disease relapse was based on aINCAT deterioration, defined as a ≥1-point increase in aINCAT compared with stage B baseline, which was confirmed at a consecutive visit after the first 1-point increase in aINCAT or not confirmed for participants with ≥2-point increase in aINCAT compared with stage B baseline. An aINCAT score of ≤−1 represented an improvement, a score of 0 represented no change and a score of ≥1 represented deterioration.
*Participants with aINCAT scores improving to stage B baseline levels or better.

^†^Of the 88 participants with clinical deterioration during stage B who enrolled into ADHERE+, one participant (receiving placebo during stage B) had restabilized at ADHERE+ baseline. This was due to a protocol deviation in which the participant had an additional visit in stage B following clinical deterioration.
Abbreviations: aINCAT: adjusted Inflammatory Neuropathy Cause and Treatment.
